# Interfacial Oxygen Octahedral Coupling-Driven Robust Ferroelectricity in Epitaxial Na_0.5_Bi_0.5_TiO_3_ Thin Films

**DOI:** 10.34133/research.0191

**Published:** 2023-07-13

**Authors:** Haojie Han, Qinghua Zhang, Wei Li, Yiqun Liu, Jiasheng Guo, Yue Wang, Qian Li, Lin Gu, Ce-Wen Nan, Jing Ma

**Affiliations:** ^1^ State Key Laboratory of New Ceramics and Fine Processing, School of Materials Science and Engineering, Tsinghua University, Beijing 100084, China.; ^2^ Beijing National Laboratory for Condensed Matter Physics, Institute of Physics, Chinese Academy of Sciences, Beijing 100190, China.

## Abstract

The oxygen octahedral rotation (OOR) forms fundamental atomic distortions and symmetries in perovskite oxides and definitely determines their properties and functionalities. Therefore, epitaxial strain and interfacial structural coupling engineering have been developed to modulate the OOR patterns and explore novel properties, but it is difficult to distinguish the 2 mechanisms. Here, different symmetries are induced in Na_0.5_Bi_0.5_TiO_3_ (NBT) epitaxial films by interfacial oxygen octahedral coupling rather than epitaxial strain. The NBT film grown on the Nb:SrTiO_3_ substrate exhibits a paraelectric tetragonal phase, while with La_0.5_Sr_0.5_MnO_3_ as a buffer layer, a monoclinic phase and robust ferroelectricity are obtained, with a remanent polarization of 42 μC cm^−2^ and a breakdown strength of 7.89 MV cm^−1^, which are the highest record among NBT-based films. Moreover, the interfacial oxygen octahedral coupling effect is demonstrated to propagate to the entire thickness of the film, suggesting an intriguing long-range effect. This work provides a deep insight into understanding the structure modulation in perovskite heterostructures and an important avenue for achieving unique functionalities.

## Introduction

ABO_3_-type perovskite oxides, consisting of the corner-sharing oxygen octahedra, offer a broad range of emergent functionalities, such as superconductivity, metal–insulator transition, ferroelectricity, and ferromagnetism, which are closely coupled to crystal symmetry [1–5]. Developing strategies to manipulate the symmetry correlated with the BO_6_ oxygen octahedral rotation (OOR) patterns has attracted extensive attention for exploring novel properties and functionalities. Apart from the widely established strain engineering for oxide symmetry modification via lattice mismatch, interfacial oxygen octahedral coupling engineering, which forces the octahedra in the film to deform or rotate (tilt) to maintain corner connectivity of oxygen octahedra at interface, has served as a degree of freedom for tuning the crystal structure and modulating physical properties such as magnetism and polarization [6–9]. However, interfacial oxygen octahedral coupling engineering remains challenging, in that a typically limited propagation thickness of less than 10 nm restricts the development of intriguing functional properties in perovskite heterostructures. In addition, the impacts of strain and interfacial oxygen octahedral coupling on film symmetry are usually coupled, resulting in challenges to distinguish them.

Na_0.5_Bi_0.5_TiO_3_ (NBT) has long been an intriguing topic among lead-free ferroelectric materials due to its macroscopic ferroelectricity and unique phase-structure flexibility, thus triggering the promising application in non-volatile memory, electromechanical device, and energy storage [10–12]. NBT is an A-site disordered perovskite material with a complex phase transition process, changing from a high-temperature cubic (*P*$m\bar{3}m$) phase to a paraelectric tetragonal (*P4bm*) phase at 540 °C, then gradually to a ferroelectric phase during 200 to 320 °C [13–15]. It has been commonly accepted that NBT exhibits the rhombohedral (*R3c*) phase at room temperature, while recent studies demonstrated the existence of monoclinic (*Cc*) phase [16–18]. Though extensive studies about the structure delineation of NBT have been reported [16–22], the complex phase structures remain open questions, mainly due to the grain boundary, grain size, and some other unwanted factors in ceramics [23–25]. Such perplexing phases play a vital role in regulating ferroelectricity, electrostriction, and other properties in NBT-based materials, and further complicate the analyses of the underlying property–structure mechanisms. In order to meet the requirement of miniaturized devices and explicitly elucidate the underlying fundamental physical mechanisms, several researchers have shed light on the development of NBT films [26–29]. However, the properties of NBT films exhibit large variations and deviations from the ferroelectric performance measured in bulk, which make them unsuitable platforms for investigating the evolution of phase structures and functionalities. Therefore, it is essential to fabricate high-quality NBT epitaxial films with superior ferroelectricity to further explore the sophisticated correlation between structure and properties.

In this work, interfacial oxygen octahedral coupling was utilized to engineer the structure symmetry and the closely related ferroelectric properties in NBT films. To minimize the strain effect, NBT films were epitaxially grown on La_0.5_Sr_0.5_MnO_3_/SrTiO_3_(001) (LSMO/STO) and Nb:SrTiO_3_(001) (NSTO) substrates, respectively. A combination of x-ray diffraction (XRD), scanning transmission electron microscopy (STEM), and optical second harmonic generation (SHG) techniques was employed to reveal the structural variation of NBT films. With LSMO as a buffer layer, the monoclinic-phase (M-phase) structure with the highest remanent polarization of 42 μC cm^−2^ among NBT-based films is stabilized in the NBT film grown on LSMO/STO substrate, while the NBT film directly grown on NSTO substrate is tetragonal-phase (T-phase) and paraelectricity. This study unequivocally reveals the specific phase modulation mechanism by interfacial oxygen octahedral coupling in NBT epitaxial film. Such strategy can be further extended to a broader class of perovskites, providing a universal guidance for designing ferroelectric devices with remarkable performances and novel functionalities.

## Results and Discussion

To demonstrate the feasibility of interfacial oxygen octahedral coupling-modulated symmetry in NBT epitaxial films, cubic single-crystal NSTO and STO (with the same lattice constant *a* = 0.3905 nm), which possess little lattice mismatch with bulk NBT (*a*_pc_ = *b*_pc_ = 0.3887 nm for M-phase, *a*_pc_ = 0.3885 nm for R-phase, and *a*_T_ = 0.3880 nm for T-phase; pc corresponds to pseudocubic), were chosen as substrates to minimize the effect of interfacial strain. For the film grown on STO, a 12-nm-thick LSMO layer (*R3c* space group in the bulk [30], *a*_pc_ = 0.3847 nm) was deposited firstly as the bottom electrode and buffer layer. The thickness of NBT films deposited on (001)-oriented LSMO/STO (denoted as NBT-L) and conductive NSTO (denoted as NBT-N) substrates is approximately 25 nm, as confirmed by high-resolution transmission electron microscopy (HRTEM, Fig. S1). It has been demonstrated that the impact of the octahedral coupling decays rapidly away from the LSMO/STO interface [6]; thus, the intrinsic oxygen octahedra structures in the 12-nm-thick LSMO rather than STO substrate should primarily affect the growth of the above NBT films.

**Fig. 1.**
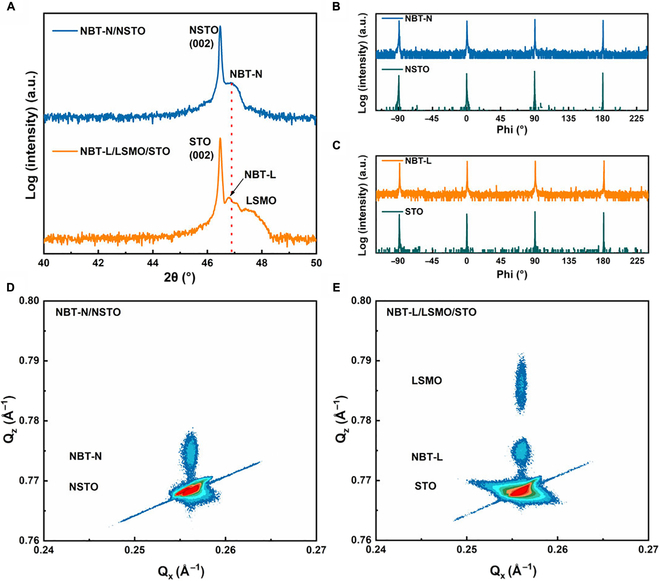
Crystal structure analysis of epitaxial NBT films. (A) XRD *θ–*2*θ* patterns around the (002) peak of NBT films. The *phi* scans of (202) planes of (B) NBT-N and NSTO, and (C) NBT-L and STO, demonstrating the in-plane 4-fold symmetric structure. Reciprocal space mappings around the STO (103) peak of (D) NBT-N/NSTO and (E) NBT-L/LSMO/STO. Q_x_ and Q_z_ represent projected directions in the reciprocal space.

*θ–*2*θ* XRD studies (Fig. S2) reveal that both NBT-L and NBT-N are epitaxial and single phase without any impurities. However, the (002) peaks display differences, i.e., single peak at 46.85° for the NBT-N film while 2 split peaks at 46.80° and 47.07° for the NBT-L film, as shown in Fig. 1A. The splitting in the (002) peak suggests the characteristic of the monoclinic structure rather than the rhombohedral structure of NBT-L, in accordance with NBT ceramics [16]. The full width at half maximum of the *ω*-scans (rocking curves) (Fig. S3) for NBT-N and NBT-L are 0.061° and 0.045°, respectively, indicating the excellent crystalline quality of both films. The epitaxial growth of NBT films was confirmed by *phi* scans of (202) planes (Fig. 1B and C) with 4-fold symmetry of films inherited from substrates. Subsequent reciprocal space mappings taken around the (103) plane of the films and substrates (Fig. 1D and E) certify that the in-plane lattice parameters of the substrates and films are identical in both NBT-N/NSTO and NBT-L/LSMO/STO systems (*a* = *b* = 0.3905 nm), but the out-of-plane lattice parameters *c* of the 2 films display slight difference, consistent with the results of *θ–*2*θ* patterns (0.3875 nm and 0.3879 nm for NBT-N and NBT-L, respectively). Comprehensively, considering the splitting of the (002) peaks and in-plane rotational symmetry, the tetragonal and monoclinic structures can be confirmed in NBT-N and NBT-L, respectively.

**Fig. 2.**
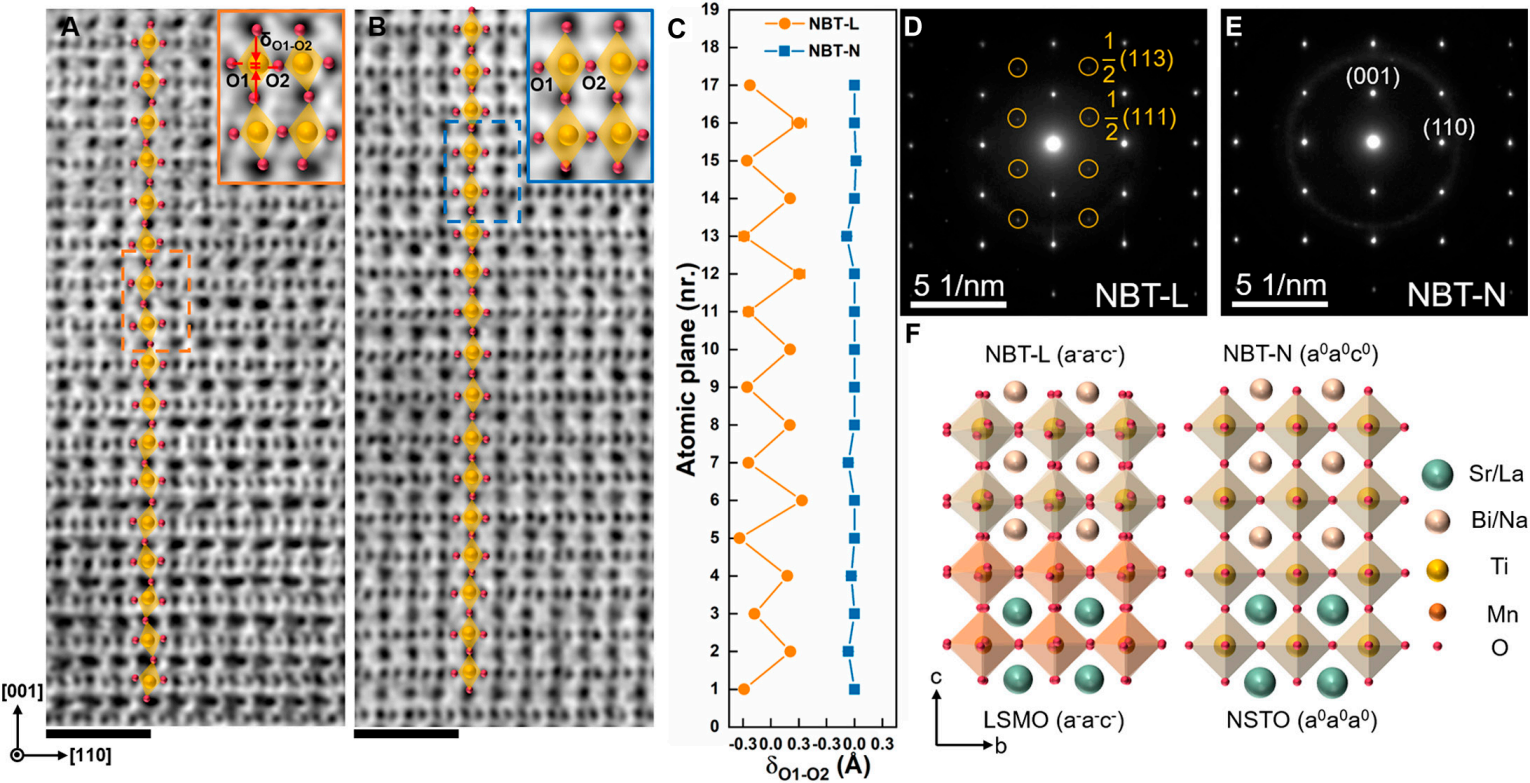
Atomic-resolution microstructure of epitaxial NBT films. Cross-sectional ABF STEM images of (A) NBT-L and (B) NBT-N films. The inset images are zoomed-in oxygen octahedra in NBT-L and NBT-N films marked in orange and blue dashed squares in (A) and (B), respectively. The scale bar is 1 nm. (C) The layer position-dependent relative shifts along [001] between O1 and O2 atoms measured from (A) and (B). The SAED patterns of (D) NBT-L and (E) NBT-N films taken from the regions marked by orange dashed squares in Fig. S1A and B, respectively. (F) Schematic models of oxygen octahedral coupling at interfaces between the NBT film and substrates.

Figure 2A and B show the cross-sectional annular bright-field (ABF) STEM images of NBT films. To effectively reveal the phase structure and OOR patterns of the epitaxial films, STEM observation was performed along the ${\left[1\bar{1}0\right]}_{\mathrm{pc}}$ zone axis. The oxygen atomic columns of the NBT-L film are clearly visible in Fig. 2A, which demonstrate the obvious oxygen octahedra tilt feature. Thus, the tilting of BO_6_ octahedra induces a relative shift (δ_O1-O2_) between the nearest 2 adjacent oxygen atoms along the [110] direction, denoted as O1 and O2. Considering that the rotation of BO_6_ octahedra about the *c* axis cannot be observed in this direction, the OOR pattern of NBT-L can be identified as a^−^a^−^c^0^ (defined by Glazer notation [31]), which is consistent with the OOR pattern of La_2/3_Sr_1/3_MnO_3_ observed along the ${\left[1\bar{1}0\right]}_{\mathrm{pc}}$ direction [32]. In contrast, the BO_6_ octahedra in the NBT-N film do not exhibit obvious tilt (as shown in Fig. 2B). Combined with the above XRD and STEM analysis, the OOR pattern of NBT-N should follow an a^0^a^0^c^0^, corresponding to the tetragonal symmetry. The precise layer position-dependent δ_O1-O2_ in both NBT films is summarized in Fig. 2C, directly reflecting the difference in the OOR. Notably, different OOR patterns can both be stabilized over a long range of 2 samples. To confirm the phase structures of NBT-L and NBT-N, selected-area electron diffraction (SAED) patterns were taken from the selected regions marked by the dashed squares in Fig. S1. Different from the NBT-N film with only regular fundamental reflections (e.g., {001} and {110}) of the pseudocubic structure (Fig. 2E), additional half-integer reflections (marked by the orange circles) $\frac{1}{2}\left\{\mathrm{ooo}\right\}$ (“o” refers to odd *hkl* indexes, e.g., $\frac{1}{2}\left\{111\right\}$ and $\frac{1}{2}\left\{113\right\}$) appear in both NBT-L (Fig. 2D) and LSMO films (Fig. S4A and B), which indicates the a^−^a^−^c^−^ OOR pattern corresponding to the typical characteristics of monoclinic symmetry [16,17]. However, considering the absence of half-integer reflections (Fig. 2E and Fig. S4D), the OOR pattern of NBT-N can be further confirmed as a^0^a^0^c^0^, with a space group of *P4mm*. Therefore, it demonstrates that with the insertion of LSMO as a buffer layer, different OOR patterns were induced, leading to the stabilization of M-phase and T-phase in NBT-L and NBT-N epitaxial films, respectively.

The formation mechanism of the different phases in NBT films can be understood as follows. Although the bulk NBT is M-phase (*Cc*)/R-phase (*R3c*) at room temperature, which possesses a^−^a^−^c^−^/a^−^a^−^a^−^ rotation with a small angle [16,25], the cubic STO and NSTO exhibit a^0^a^0^a^0^ pattern without octahedral rotation. In the heterosymmetric interface, interfacial oxygen octahedral coupling effects offer the interfacially engineered oxygen environment, which leads to the change of B–O–B bond angle and the deformation of oxygen octahedral to achieve minimal oxygen octahedral mismatch between epitaxial film and substrate [33]. As shown in Fig. 2F, for the NBT film directly epitaxially grown on NSTO substrate, the interfacial oxygen positions are strongly restricted by the connection of octahedra at the interface, resulting in the stabilization of the T-phase structure with the Ti–O–Ti bond angle of ~180° and the OOR of a^0^a^0^c^0^ in NBT-N. However, with LSMO as a buffer layer, the a^0^a^0^a^0^ rotation of STO can only affect the OOR of LSMO close to the LSMO/STO interface within about 8 u.c. [6]. Thus, in the case of the 12-nm-thick LSMO, the NBT will only be subjected to the OOR pattern of LSMO (a^−^a^−^c^−^) at the NBT/LSMO interface, further facilitating the formation of M-phase NBT with a^−^a^−^c^−^ rotation. Thus, through the interfacial oxygen octahedral coupling engineering, the structure symmetry of the epitaxial NBT films can be explicitly controlled. Furthermore, according to the single-phase structure in NBT-L and NBT-N confirmed by XRD and SAED, the interfacial oxygen octahedral coupling effect can stabilize the M- and T-phase NBT film throughout the entire thickness of 25 nm, which is probable due to the small energy differences among different phases of NBT [34,35]. Therefore, considering that the symmetry constraints are enforced to every subsequent layer, for the films with the smaller energy differences between the metastable phase and equilibrium phase, the initiated novel symmetry is likely to sustain in a longer thickness range.

**Fig. 3.**
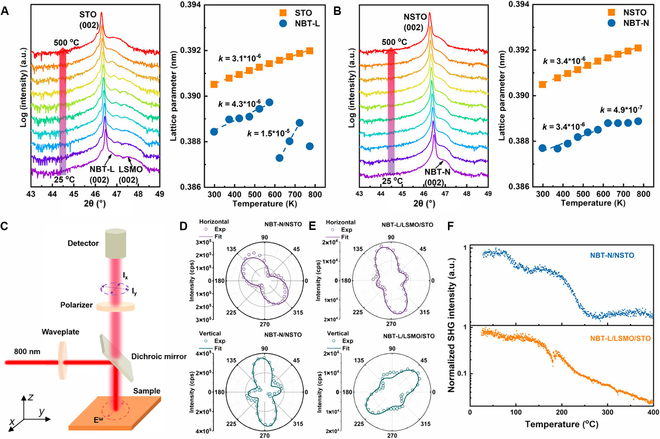
Structural symmetry characterization and phase evolutions of NBT films. In situ XRD results and the evolution of *c*-axis parameters of both the substrates and the films of (A) NBT-L/LSMO/STO and (B) NBT-N/NSTO around the STO (002) or NSTO (002) peaks at different temperatures during vacuum annealing. (The *θ–*2*θ* patterns were collected at 25 °C and 100 to 500 °C with the temperature interval of 50 °C. *k* represents the thermal expansion coefficient with the unit of K^−1^.) (C) Schematic configuration of the optical SHG experiment. SHG patterns under both horizontal and vertical modes in (D) NBT-N/NSTO and (E) NBT-L/LSMO/STO. Circles represent experimental data; lines represent fittings. (F) Temperature-dependent SHG intensity of NBT-N/NSTO and NBT-L/LSMO/STO.

In order to trace the phase transition and further verify the phase structure of the NBT films, in situ XRD measurements were performed during heating and the *c*-axis parameters and the thermal expansion coefficients were further calculated based on the in situ XRD results. Figure 3A and B display the corresponding *θ–*2*θ* patterns around the (002) reflection peaks from 25 to 500 °C. As the annealing proceeds, the (002) peaks of the substrate and the film shift toward lower angles due to thermal expansion, which is reflected in the increased *c*-axis parameters with the thermal expansion coefficient of about 3 to 4 ppm K^−1^. However, the initial (002) peak of NBT-L gradually fades away above 300 °C, while a new peak emerges at a higher angle position and the thermal expansion coefficient changes to 15 ppm K^−1^, which demonstrates the end of phase transition and a new phase appeared in NBT-L. According to the previous report [13] and the above structural analyses, this phase transition can be attributed to the M–T transition of NBT. As the temperature increased above 450 °C, the peaks of NBT-L and LSMO merge into one peak with the *c*-axis parameters of about 0.3878 nm. This should be ascribed to the gradual oxygen loss in LSMO [36], which leads to the structural disruption of NBT-L, as evidenced in Fig. S5. In contrast, the peak position of the NBT-N film continues to shift toward lower angles until 500 °C, but the thermal expansion coefficient changes from 3.4 ppm K^−1^ to 4.9 ppm K^−1^ around 300 °C (Fig. 3B), which indicates that the NBT-L film exhibits a different phase transition process from NBT-N film during heating.

Optical SHG is a sensitive probe to acquire abundant information about local crystal symmetry, domain distribution, phase transition process, etc. [37–40]. As schematically shown in Fig. 3C, the rotational anisotropy of the optical SHG intensity is examined with a normal-incidence configuration, in which the SHG signal is collected in a reflecting geometry and the incident light can be focused to a spot of ~1 μm for polar-domain imaging, i.e., scanning SHG microscopy [40]. In this configuration, the incident polarized light beam provides only the electrical field components in the *x–y* plane (defined in Fig. 3C) and only the polarization in the *x–y* plane (in-plane) can be collected through the polarizer. Thus, it effectively probes the crystal symmetry of polar materials with spontaneous polarization (*P_s_*) variants in the *x–y* plane. The in-plane anisotropic SHG patterns of NBT-L and NBT-N (Fig. S6) show different preferred orientations of domains, which imply different crystal structures. In addition, horizontal and vertical mode measurements, corresponding to the cases with the analyzer parallel and perpendicular to the polarization direction of the incident beam, respectively, were performed to determine *P_x_* and *P_y_* to further illustrate the symmetry of the films. For NBT-N, one double lobe in horizontal mode and 2 double lobes in vertical mode are observed in Fig. 3D, which indicates the presence of tetragonal phase [41]. In contrast, for NBT-L, both horizontal and vertical modes have 2 double lobes (Fig. 3E), consistent with the feature of a monoclinic phase [8]. The fitting of SHG data further verify the M- and T-phase symmetry of NBT-L and NBT-N, respectively (details in the Supplementary Materials). In addition, the observed patterns cannot be numerically reproduced when a single domain model is considered, and the agreement between the SHG signal (data points) and the simulation (line) is only achieved when considering multi-domain models in both cases.

Figure 3F displays the temperature-dependent SHG signals of NBT-L and NBT-N films. The decrease of SHG intensity demonstrates that the proportion of polar domains decreases with increasing temperature for both samples [42]. The small hump at 100 to 150 °C is ascribed to the increased domain walls induced by thermally enhanced local disorder in the multi-domain structure [43]. The SHG intensity of NBT-L displays 2 humps as temperature increases to 190 and 320 °C, yet not presented in NBT-N. These features are sensitive indicators of the M–T phase transition in the temperature range [21], consistent with the in situ XRD results (Fig. 3A and B). In addition, the SHG intensity of NBT-N and NBT-L drops to minimum values above 260 and 400 °C, respectively, indicating a tetragonal–cubic phase transition. Therefore, the SHG results further evidence the monoclinic symmetry for NBT-L and tetragonal symmetry for NBT-N.

**Fig. 4.**
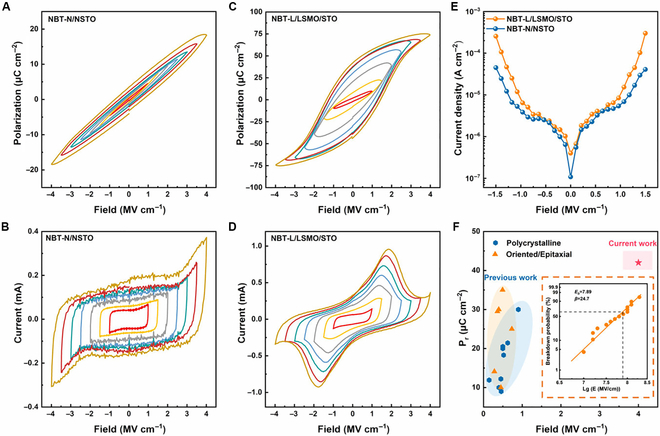
Ferroelectricity characterization of NBT films. *P*–*E* loops at different electric fields of (A) NBT-N/NSTO and (C) NBT-L/LSMO/STO. *I–V* curves at different electric fields of (B) NBT-N/NSTO and (D) NBT-L/LSMO/STO. (E) Leakage current densities of the NBT-N and NBT-L films. (F) Comparison of remanent polarization in NBT-based thin films between the current work and those reported previously (the inset is the 2-parameter Weibull distribution analysis of the characteristic breakdown field of the NBT-L film) [27,28,48–60].

Due to the different structure symmetries, the ferroelectric properties of NBT-L and NBT-N films, which are closely related to the structure, must also be different. Piezoresponse force microscopy (PFM) images of 2 samples are present in Fig. S7. The labyrinthine domains and local phase hysteresis loops in the NBT-L film demonstrate the typical characteristic of ferroelectric phase NBT [44]. On the contrary, the paraelectric nature is observed in the NBT-N film. Figure 4A and C display the polarization versus electrical field (*P*–*E*) loops of both films, further demonstrating the different ferroelectricity in 2 samples. Remarkably, with almost the same film thickness of ~25 nm, NBT-L exhibits a typical ferroelectric hysteresis while the paraelectric characteristic can be observed in NBT-N. By applying an electric field of 4 MV cm^−1^, the remanent polarization (*P_r_*) of NBT-L reaches approximately 42 μC cm^−2^, approaching the bulk value of M-phase NBT [22]. Two obvious ferroelectric switching current peaks appear in current versus voltage (*I–V*) curves of NBT-L (Fig. 4D), while only the linear polarization current and leakage current contribution are displayed in NBT-N (Fig. 4B). The coercive field (*E_c_*) of NBT-L is estimated to be around 1.4 MV cm^−1^, much larger than that of the bulk NBT (about 50 to 80 kV cm^−1^), which could be ascribed to the thickness effect [45,46]. Additionally, the leakage current densities of both samples, shown in Fig. 4E, are at low levels, which excludes the possible artifacts of ferroelectricity caused by the leakage current. It is also worth mentioning that, although plenty of NBT-based films have been investigated (detailed data in Table S1), the NBT-L film in this work exhibits the highest *P_r_* of 42 μC cm^−2^, which approached the theoretical value in NBT ceramics [22], and high electrical breakdown strength (*E_b_*) of 7.89 MV cm^−1^ (Fig. 4F). Such high *P_r_* and large *E_b_* give credit to the high crystal quality and well epitaxial orientation of the pure M-phase structure in NBT-L, which importantly extends the performance boundary of NBT-based thin films. Meanwhile, considering the <001> epitaxial orientation of NBT-L, the present polarization is higher than the theoretical value for R-phase NBT of ~23 μC cm^−2^ in this direction [14], which further confirmed the existence of the M-phase (*Cc*) structure. All results above demonstrate the remarkable ferroelectric properties in NBT-L films, ensuring their tremendous potential in high-performance miniaturized ferroelectric devices. Moreover, the correlation between ferroelectricity and phase structure is unequivocally revealed in epitaxial NBT films, demonstrating the critical role of the interfacial oxygen octahedral coupling effect.

## Conclusion

In summary, interfacial oxygen octahedral coupling-driven symmetry manipulation is realized in NBT films grown on NSTO and LSMO/STO substrates. With the same epitaxial strain, the a^−^a^−^c^−^ OOR pattern of LSMO promotes the formation of M-phase NBT, instead of T-phase with a^0^a^0^c^0^ rotation inherited from the NSTO or STO substrate. The phase evolution process in M-phase and T-phase NBT films was further illustrated, thus facilitating the development of high-performance NBT-based materials. Remarkably, the M-phase NBT film exhibits robust ferroelectricity with a remanent polarization of 42 μC cm^−2^ and a breakdown strength of 7.89 MV cm^−1^, which are the highest record among NBT-based films. This result unequivocally reveals that interfacial oxygen octahedral coupling engineering can provide a new degree of freedom for manipulating phase structures and ferroelectricity in NBT as a long-range effect, and is expected to provide an important avenue for manipulating the structures and design multifunctional perovskite oxides for a myriad of applications.

## Materials and Methods

### Film preparation

NBT ceramic target was sintered by conventional solid-state reaction. High-purity Bi_2_O_3_, TiO_2_, and Na_2_CO_3_ powders were mixed according to the designed stoichiometric proportions, ball-milled for 12 h and then calcined at 850 °C for 3 h. To compensate for the volatilization of Bi and Na during target sintering and film fabrication, 5 mol% Bi and 10 mol% Na were added in excess. The calcined powders were then compacted into disks at 8 MPa for 5 min. Finally, the target was sintered at 1,100 °C for 3 h.

The NBT-L thin film was fabricated on single-crystalline (001)-oriented STO substrates using pulsed laser deposition, with the LSMO film grown as the bottom electrode. The NBT-N thin film was fabricated on (001)-oriented 0.7 wt.% Nb-doped STO (Nb: STO, NSTO) with the same growth condition of NBT-L. For deposition of NBT and LSMO films, a KrF excimer laser, with a wavelength of 248 nm, was used. The NBT-L and NBT-N thin films’ growth was carried out at a heater temperature of 700 °C in a dynamic oxygen pressure of 0.2 mbar with a laser fluence of 0.9 J cm^−2^ and a laser repetition rate of 2 Hz from the same ceramic target. The LSMO thin film growth was carried out at the same temperature and oxygen pressure with NBT, with a higher laser fluence of 1.2 J cm^−2^ and a higher laser repetition rate of 5 Hz. After deposition, the films were annealed in an oxygen-enriched environment (200 mbar) for 20 min at the growth temperature, and then slowly cooled down to room temperature at a rate of 5 °C min^−1^. The sample thickness was controlled by the growth time and calibrated by x-ray reflection.

### Structural characterization

The crystal structure and epitaxial quality were characterized using an x-ray diffractometer (X'pert Pro2, PANalytical) with Cu Kα1 radiation (λ = 1.5406 Å). For the in situ XRD measurement, the samples were heated from 25 to 500 °C in a vacuum chamber (10^−3^ mbar). The temperature was ramped at a rate of 20 °C min^−1^ and maintained for 10 min at each test temperature for stabilization. Cross-sectional TEM samples were prepared using Ga^+^ ion milling (Zeiss FIB). An ARM-200CF (JEOL) TEM equipped with double spherical aberration (Cs) correctors was used for structure analysis of the NBT thin films. The ABF STEM images were collected at an operating voltage of 200 kV and an acceptance angle of 12 to 24 mrad.

The polarization-dependent and temperature-dependent SHG measurements were performed by using a 800-nm laser (80 MHz, 35 fs) generated from a Ti:sapphire mode-locking femtosecond laser (MaiTai SP, Spectra-Physics) as the fundamental laser beam. The generated SHG signals were then collected by a photomultiplier tube with an optical filter to rule out 800-nm reflected light. For temperature-dependent SHG measurements, a closed heating stage with a sapphire window was used. The temperature was ramped at a rate of 5 °C min^−1^ controlled by a proportional integral derivative controller with a cycled water-cooling system. During the measurement, the focal spot and the morphology features on the films were checked every 5 min to keep the incident power and the probing region unchanged.

### PFM and electrical measurements

PFM experiments were performed using an Infinity Asylum Research AFM in ambient conditions at room temperature with commercial conductive tips (PPP-EFM, Nanosensors). The PFM signal was collected at the contact resonance frequency with an a.c. tip bias of 1 V. Electrical measurements were carried out with a planar capacitor structure with LSMO or NSTO bottom electrodes and Au top electrodes. Circular Au electrodes of ~60 nm in thickness were sputtered through a stainless-steel shadow mask with holes of ~150 μm in diameter. Note that the real electrode diameter would be slightly larger than 150 μm due to the diffusion during sputtering. The real electrode size (~160 μm) was measured and calibrated with optical microscopy before electrical measurements. Ferroelectric properties were characterized on a Sawyer-Tower circuit (Precision Multiferroic II, Radiant Technologies). Polarization–electric field (*P*–*E*) loops and current loops were collected using double bipolar triangular voltage waves with a frequency of 5 kHz. The leakage currents were obtained by applying a multistep DC voltage, with a soak time of 100 ms and a measurement time of 100 ms for each step.

The dielectric breakdown field of NBT-L was derived from the 2 parameter Weibull distribution function [47]:

$$P\left(E_{i}\right)=1-\exp\left(-{\left(E_{i}/E_{b}\right)}^{\beta }\right)$$ (1)

where *E_i_* is the measured breakdown field of *i*th data arranged in ascending number, *P*(*E_i_*) is the cumulative probability of electric breakdown at *E_i_*, *E_b_* is the statistical Weibull characteristic breakdown field at which *P*(*E_i_*) equals 63.2%, and the Weibull parameter *β* evaluates the distribution of *E_i_*. The 2 parameters, *E*_b_ and *β*, can be extrapolated from the linear fitting result of Eq. 2. Fifteen *E_i_* values were collected from different samples for the analysis. The high Weibull moduli can reflect good homogeneity.

$$\text{lg}\left(-\ln\left(1-P\left(E_{i}\right)\right)\right)=\gamma \mathrm{lg}\left(E_{i}/E_{b}\right)$$ (2)

## Data Availability

All data needed to evaluate the conclusions in the paper are present in the paper and/or the Supplementary Materials. Supplementary Materials are available in the online version of the paper. Reprint and permission information is available online.

## Supplementary Materials

Supplementary 1 Fitting details of SHG results Extended verification experiment Fig. S1. Cross-sectional HRTEM images of (A) NBT-L/LSMO/STO and (B) NBT-N/NSTO. Fig. S2. XRD *θ*–2*θ* patterns with the broad angle region of NBT-N/NSTO and NBT-L/LSMO/STO. Fig. S3. Rocking curves for the (002) peaks of NBT-N and NBT-L films. Fig. S4. (A) Cross-sectional HRTEM images of NBT-L/LSMO/STO. Fig. S5. XRD *θ*–2*θ* patterns around the (002) peaks of film before and after annealing at 500 °C for (A) NBT-L/LSMO/STO and (B) NBT-N/NSTO. Fig. S6. The in-plane anisotropic SHG patterns of (A) NBT-L and (B) NBT-N. Fig. S7. PFM images of the NBT-L and NBT-N films. Fig. S8. Ferroelectricity characterization of the NBT film with the thickness of 25 nm grown on LSMO (3 u.c.)/NSTO. Table S1. Thickness and ferroelectric properties of NBT-based thin films.
