# Supplementary material for: Interfacial Oxygen Octahedral Coupling-Driven Robust Ferroelectricity in Epitaxial Na0.5Bi0.5TiO3 Thin Films
Source: Research (Wash D C). 2023 Jul 13;6:0191. doi: 10.34133/research.0191 (PMC10351392; doi:10.34133/research.0191)
Supplement: Supplementary 1 — Fitting details of SHG results Extended verification experiment Fig. S1. Cross-sectional HRTEM images of (A) NBT-L/LSMO/STO and (B) NBT-N/NSTO. Fig. S2. XRD θ–2θ patterns with the broad angle region of NBT-N/NSTO and NBT-L/LSMO/STO. Fig. S3. Rocking curves for the (002) peaks of NBT-N and NBT-L films. Fig. S4. (A) Cross-sectional HRTEM images of NBT-L/LSMO/STO. Fig. S5. XRD θ–2θ patterns around the (002) peaks of film before and after annealing at 500 °C for (A) NBT-L/LSMO/STO and (B) NBT-N/NSTO. Fig. S6. The in-plane anisotropic SHG patterns of (A) NBT-L and (B) NBT-N. Fig. S7. PFM images of the NBT-L and NBT-N films. Fig. S8. Ferroelectricity characterization of the NBT film with the thickness of 25 nm grown on LSMO (3 u.c.)/NSTO. Table S1. Thickness and ferroelectric properties of NBT-based thin films. [file research.0191.f1.docx]

Supplementary Materials for

**Interfacial Oxygen Octahedral Coupling Driven Robust Ferroelectricity in Epitaxial Na_0.5_Bi_0.5_TiO_3_ Thin Films**

Haojie Han^1^, Qinghua Zhang^2^, Wei Li^1^, Yiqun Liu^1^, Jiasheng Guo^1^, Yue Wang^1^, Qian Li^1^, Lin Gu^1^, Ce-Wen Nan^1^, Jing Ma^1^*

*^1^State Key Laboratory of New Ceramics and Fine Processing, School of Materials Science and Engineering, Tsinghua University, Beijing 100084, China.*

*^2^ Beijing National Laboratory for Condensed Matter Physics, Institute of Physics, Chinese Academy of Science, Beijing 100190, China.*

*****Address correspondence to: Jing Ma; ma-jing@tsinghua.edu.cn

**Supplementary Text**

Fitting details of SHG results

In this section, we present a detailed analysis procedure for the SHG anisotropy patterns by considering several alternative point group symmetries of NBT films. SHG is an example of a second nonlinear optical process and its nonlinear polarization can be expressed as:

$P_{s}^{2\omega}=\left(\begin{matrix} P_{1} \\ P_{2} \\ P_{3} \end{matrix}\right)=d_{ijk}\left(\begin{matrix} E_{1}^{2} \\ E_{2}^{2} \\ E_{3}^{2} \\ 2E_{2}E_{3} \\ 2E_{1}E_{3} \\ 2E_{1}E_{2} \end{matrix}\right)$ (1)

The *d_ijk_* is the nonlinear optical tensor, where the crystallography coordinate (X_1_, X_2_, X_3_) is established to denote the orientation of nonlinear optical tensor (*d_ijk_*) coordinates of each individual domain variants. The monoclinic (M-phase) ferroelectric films exhibit the nonlinear optical tensors *d_ijk_* as:

$d_{ijk}^{R}=\left[\begin{matrix} \begin{matrix} d_{11} \\ 0 \\ d_{31} \end{matrix} & \begin{matrix} d_{12} \\ 0 \\ d_{32} \end{matrix} & \begin{matrix} \begin{matrix} \begin{matrix} d_{13} \\ 0 \\ d_{33} \end{matrix} & \begin{matrix} 0 \\ d_{24} \\ 0 \end{matrix} \end{matrix} & \begin{matrix} d_{15} \\ 0 \\ d_{35} \end{matrix} & \begin{matrix} 0 \\ d_{26} \\ 0 \end{matrix} \end{matrix} \end{matrix}\right]$ (2)

The tetragonal (T-phase) ferroelectric films exhibit nonlinear optical tensors *d_ijk_* as:

$d_{ijk}^{T}=\left[\begin{matrix} \begin{matrix} 0 \\ 0 \\ d_{31} \end{matrix} & \begin{matrix} 0 \\ 0 \\ d_{31} \end{matrix} & \begin{matrix} \begin{matrix} \begin{matrix} 0 \\ 0 \\ d_{33} \end{matrix} & \begin{matrix} 0 \\ d_{15} \\ 0 \end{matrix} \end{matrix} & \begin{matrix} d_{15} \\ 0 \\ 0 \end{matrix} & \begin{matrix} 0 \\ 0 \\ 0 \end{matrix} \end{matrix} \end{matrix}\right]$ (3)

The two experimentally accessible components of the SHG intensity can be expressed as:

$I_{x}^{2\omega}=I_{X_{1}}^{2\omega}\propto(P^{2\omega}\bullet A_{x})^{2}$ (4)

$I_{y}^{2\omega}=I_{X_{2}}^{2\omega}\propto(P^{2\omega}\bullet A_{y})^{2}$ (5)

where *A_i_* corresponds to the orientation of the output analyzer in position *i* (expressed in the domain coordinate system).

Considering the contribution of multi-domains to the SHG in NBT films, it is reasonable to assume that each nanometer-sized domain contributes to the SHG responses coherently as $I^{2\omega}\propto(P_{1}+P_{2})^{2}$ (6)

Here, *P_1_* and *P_2_* imply the induced SH polarizations of each nanodomain when only two types of domains are considered.

For NBT-N sample, the fitting functions could be expressed as:

 (7)

 (8)

For NBT-L, the fitting functions could be expressed as:

 (9)

 (10)

The fitting curves of *I_H/V_* and *I_C_* are present in Figure 3(d), 3(e) and Figure S6, respectively.

Extended verification experiment

To further confirm the different ferroelectricity induced by the interfacial oxygen octahedra coupling, a 3 u.c. thick LSMO was utilized as the buffer layer on NSTO substrate, in which the successive a^0^a^0^c^0^ rotation inherited from the NSTO substrate is not relaxed in 3 u.c. As expected, NBT (25 nm)/LSMO (3 u.c.)/NSTO presents the similar paraelectric behavior to NBT-N/NSTO and no obvious ferroelectric switching peaks are observed (Figure S8). Therefore, the OOR patterns of NBT are strongly correlated with the underneath LSMO electrode.


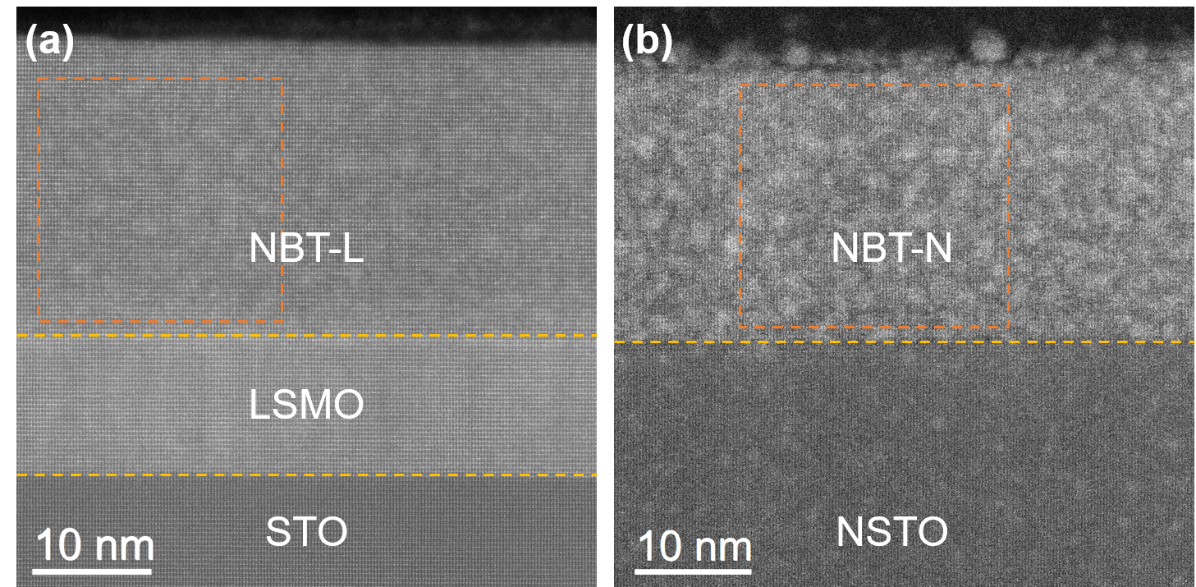


**Figure S1.** Cross-sectional high-resolution transmission electron microscopy images of (a) NBT-L/LSMO/STO and (b) NBT-N/NSTO.





**Figure S2.** XRD θ-2θ patterns with broad angle region of NBT-N/NSTO and NBT-L/LSMO/STO.





**Figure S3.** Rocking curves for (002) peaks of NBT-N and NBT-L films.


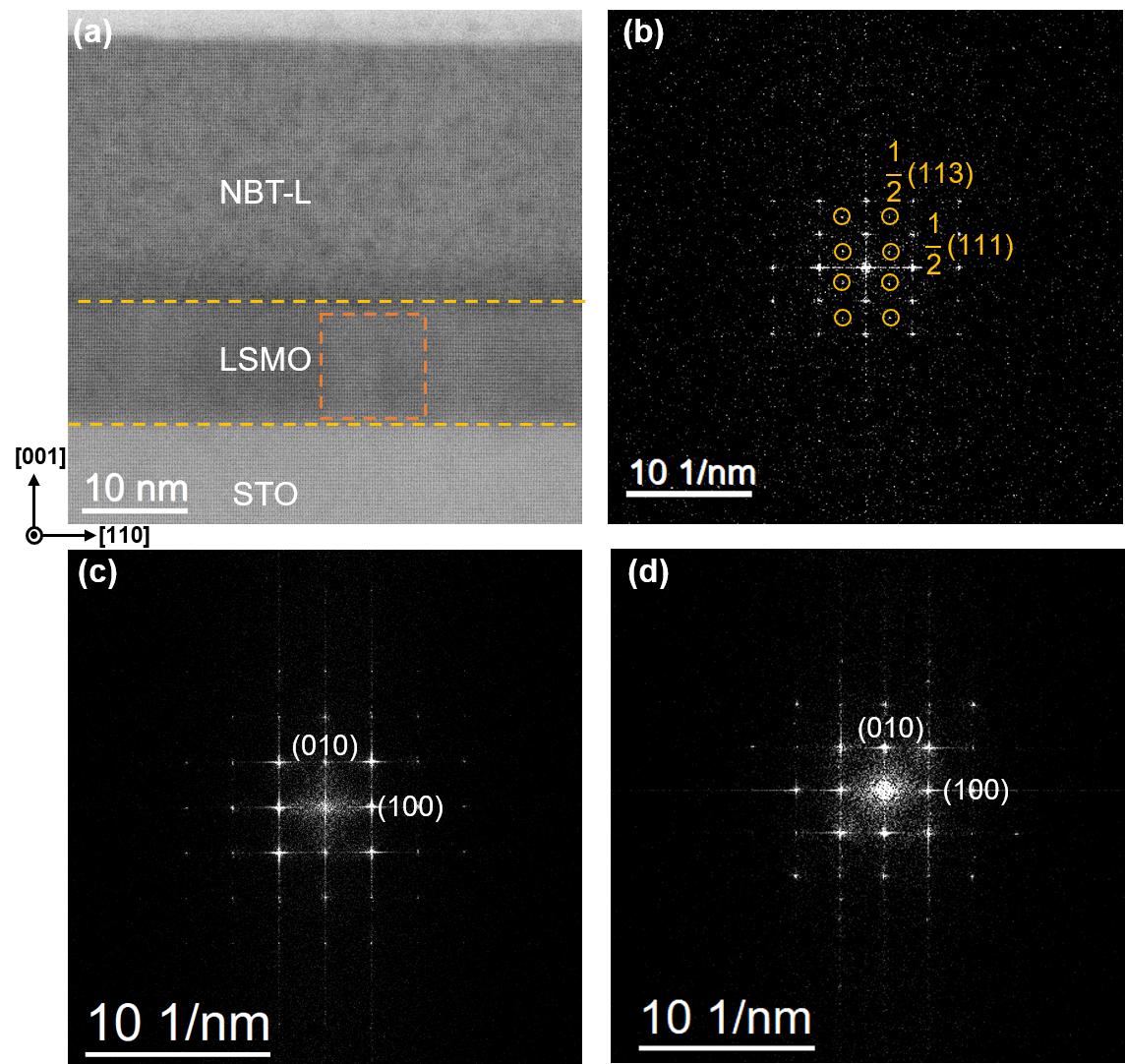


**Figure S4.** (a) Cross-sectional HRTEM images of NBT-L/LSMO/STO. (b) The FFT pattern taken from the region marked by orange dashed squares in (a). The FFT patterns along [100] zone axes of (c) NBT-L and (d) NBT-N.


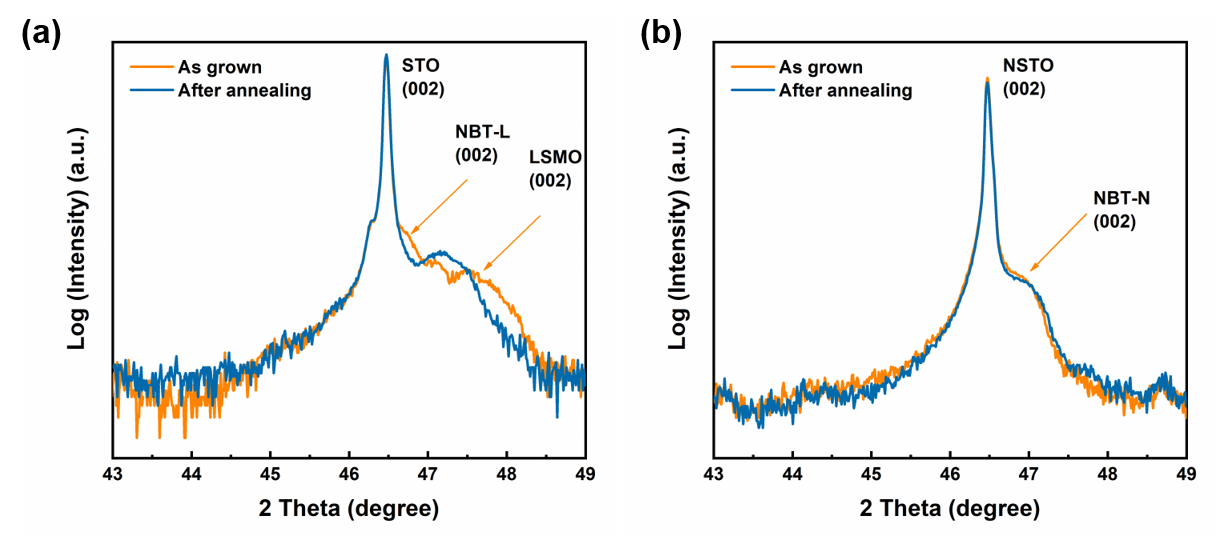


**Figure S5.** XRD *θ-*2*θ* patterns around the (002) peaks of film before and after annealing at 500℃ for (a) NBT-L/LSMO/STO and (b) NBT-N/NSTO. After annealing, the peaks of NBT-L and LSMO merge into one peak, while the peak of NBT-N keeps at the same angle position.


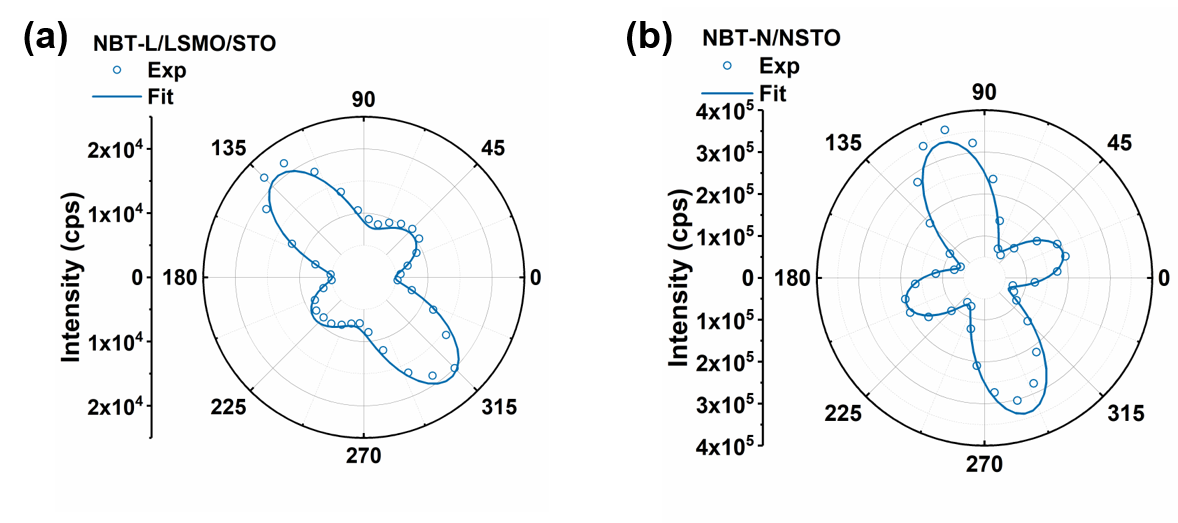


**Figure S6.** The in-plane anisotropic SHG patterns of (a) NBT-L and (b) NBT-N. The polarization of the incident light was rotated by an angle φ ranging from 0° to 360° with the concurrent rotation of the polarizer, denoted as couple mode. The different preferred orientations of domains imply different crystal structures between NBT-L and NBT-N films.


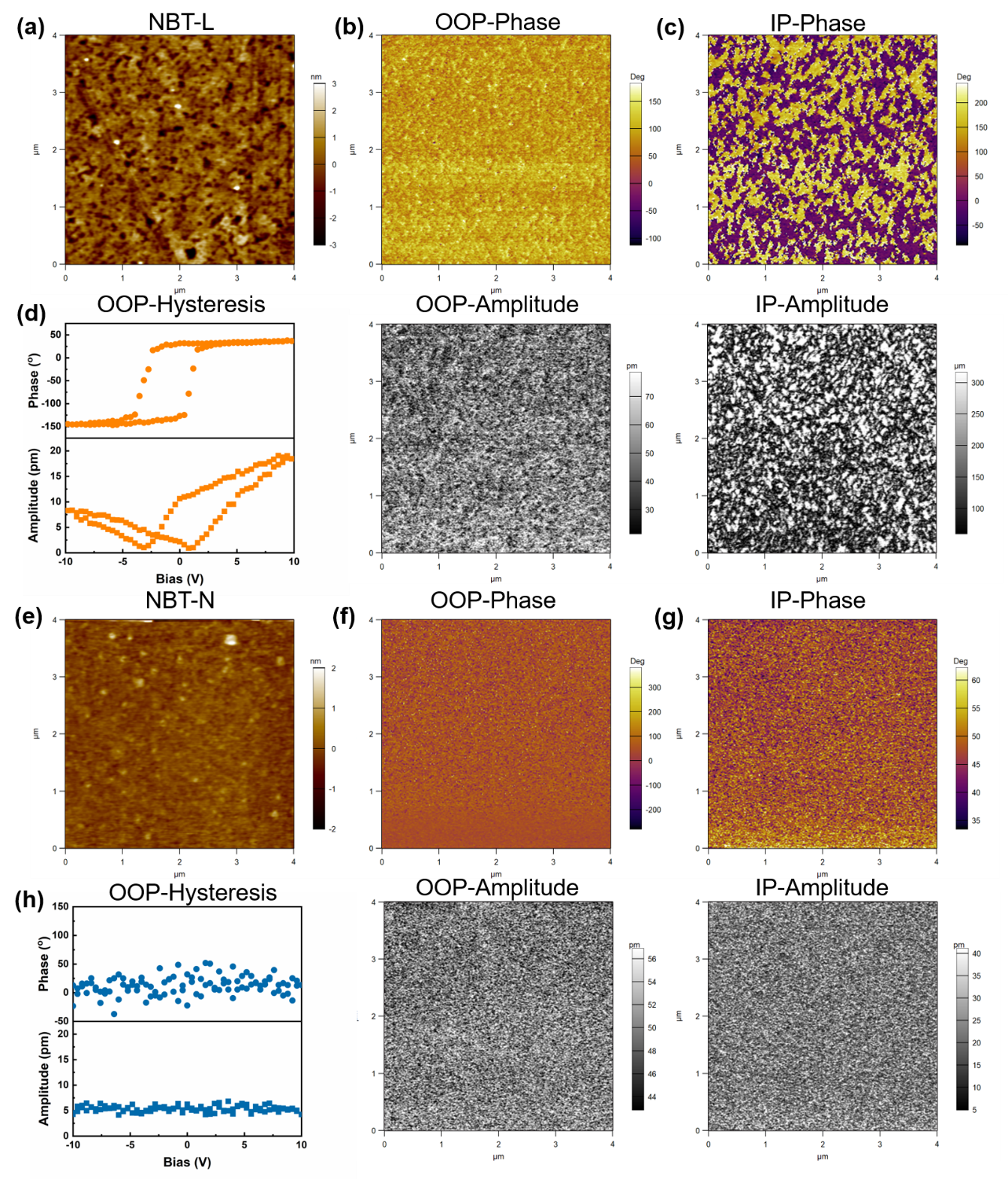


**Figure S7.** PFM images of NBT-L and NBT-N film. (a, e) Surface morphology, (b, f) out-of-plane PFM phase and amplitude, (c, g) in-plane PFM phase and amplitude, (d, h) local phase hysteresis loops and amplitude butterfly curves obtained from out-of-plane signal images of the NBT-L and NBT-N films.


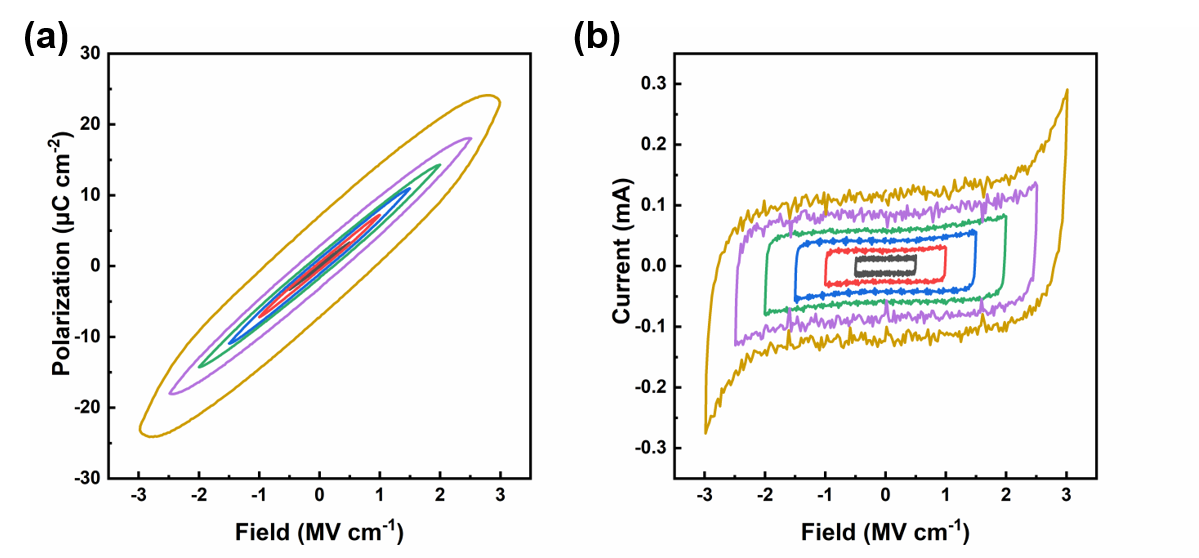


**Figure S8.** Ferroelectricity characterization of NBT film with the thickness of 25 nm grown on LSMO (3 u.c.)/NSTO. (a) *P-E* loops and (b) *I-V* curves at different electric fields. Here NSTO substrate is used to act as the bottom electrode, as the 3 u.c. LSMO is not conductive enough.

**Table S1.** Thickness and ferroelectric properties of NBT-based thin films.

| **Materials (the content of NBT >0.5)** | **Thickness [nm]** | **Electrical field [kV cm^-1^]** | **Coercive field [kV cm^-1^]** | **Remanent polarization [µC cm^-2^]** | **Ref.** |
| --- | --- | --- | --- | --- | --- |
| Na_0.5_Bi_0.5_TiO_3_ (polycrystalline) | 350 | ~457 | 90 | 9 | [29] |
| Na_0.5_Bi_0.5_TiO_3_ (polycrystalline) | 400 | 500 | 150 | 20.5 | [47] |
| Na_0.5_Bi_0.5_TiO_3_ (polycrystalline) | 200 | 500 | 160 | 20 | [53] |
| Na_0.5_Bi_0.5_TiO_3_ (polycrystalline) | 400 | 625 | 187 | 21.42 | [50] |
| Na_0.5_Bi_0.5_TiO_3_ (polycrystalline) | ~170 | 147 | 37.9 | 11.9 | [59] |
| 0.85Bi_0.5_Na_0.5_TiO_3_-0.15Bi_0.5_K_0.5_TiO_3_-0.03SrZrO_3_ (polycrystalline) | 250 | ~450 | 120.5 | 12.2 | [49] |
| Bi_0.5_Na_0.5_TiO_3_-BaTiO_3_-BiInO_3_ (polycrystalline) | 400 | 400 | ~50 | 10 | [52] |
| Pr^3+^-doped Na_0.5_Bi_0.5_TiO_3_ (polycrystalline) | 400 | 520 | 94 | 18.3 | [55] |
| 0.74Bi_0.5_Na_0.5_TiO_3_-0.06BaTiO_3_-0.2SrTiO_3_ (polycrystalline) | 100 | 900 | ~350 | ~30 | [51] |
| Na_0.5_Bi_0.5_TiO_3_ (<110>) | 450 | 250 | 85 | 14.1 | [54] |
| Na_0.5_Bi_0.5_TiO_3_ (<110>) | 400 | ~437 | ~100 | 35 | [28] |
| Bi_0.5_Na_0.5_TiO_3_-BaTiO_3_-5 mol% Mn (<001>) | 160 | ~650 | 150 | 25 | [48] |
| Ce^4+^ and La^3+^ doped (Bi_0.5_Na_0.5_)_0.94_Ba_0.06_TiO_3_ (<001>) | 380 | ~320 | 74 | 29.5 | [57] |
| (Bi,Na)TiO_3_-(Bi,K)TiO_3_-BaTiO_3_ (<001>) | 350 | 350 | 95 | 30 | [56] |
| (Na_0.5_Bi_0.5_)_0.94_Ba_0.06_TiO_3_ (<001>) | 250 | 400 | ~100 | ~10 | [58] |
| Na_0.5_Bi_0.5_TiO_3_ (<001>) | 25 | 4000 | 1400 | 42 | This work |

All references mentioned in Table S1 are those references quoted in the main text.
